# Craniofacial height in relation to cross-sectional maxillary and mandibular morphology

**DOI:** 10.1186/s40510-017-0187-8

**Published:** 2017-10-23

**Authors:** Anna Klinge, Karin Becktor, Christina Lindh, Jonas P Becktor

**Affiliations:** 1 Department of Oral and Maxillofacial Surgery and Oral Medicine, Faculty of Odontology, Malmö University, SE-205 06 Malmö, Sweden; 2 Clinic for Orthodontics and Oral Surgery, Strandvejen 116A, 2900 Hellerup, Copenhagen Denmark

**Keywords:** Craniofacial development, Treatment planning, Treatment timing, Cephalometrics, Biological basis of orthodontics

## Abstract

**Background:**

In order to gain a better understanding of how growth of the alveolar bone is linked to the vertical development of the face, the purpose of this study was to investigate if there is an association between the cross-sectional morphology of the maxillary and mandibular bodies with the craniofacial height, using images from cone beam computed tomography (CBCT).

**Methods:**

From 450 pre-treatment CBCT scans, 180 were selected to be included in the study. Lateral head images were generated from the CBCT scans and were used to categorise subjects into three groups based on their vertical craniofacial height. Cross-sectional images from CBCT volumes were reformatted of the maxillary and mandibular bodies at five locations in the maxilla and five in the mandible. Each image was measured at one height and two width measurements. Statistical analysis performed was the one-way analysis of variance with a Tukey post hoc test. A significance level of 5% was used in all comparisons.

**Results:**

Patients with large vertical craniofacial height had a significantly higher cross-sectional area both in the maxilla and in the mandible. In the same group, the cross-sectional area was significantly thinner in the mandible compared with the other two groups, especially in the anterior region.

**Conclusions:**

This study further highlights the close relationship between craniofacial height and alveolar bone dimensions and contributes with important knowledge for planning and follow-up of comprehensive dental- and orthodontic treatments.

## Background

Craniofacial growth is a complex process, and understanding the factors involved is important in connection with orthodontic treatment of children and adolescents. The growth of the craniofacial complex can be divided into four different components: (1) growth of the cranial base, (2) growth of the maxilla, (3) growth of the mandible and (4) growth of the dentoalveolar bone [1].

In the postnatal growth period, growth in the spheno-occipital synchondrosis cartilage and the cartilage of the condyle has a large influence on the vertical development of the face, and adaptive growth of the maxillary sutures and growth of the dentoalveolar bone will fill out the face [2]. The link between the predominately genetically determined growth of these two cartilages and growth of the dentoalveolar bone is also known as the dentoalveolar compensatory mechanism [3, 4]. The factors that are responsible for this dentoalveolar mechanism are not fully understood. However, it has been demonstrated that growth of the dentoalveolar bone is taking place in connection with tooth eruption and is accordingly dependent on a normal eruption process [4].

The vertical development of the face has a significant impact on the direction and magnitude of tooth eruption [5]. There is a noticeable variation in the eruption rate of the teeth, with a peak in eruption velocity at the time of the maximum pubertal rate of growth in body height [6]. Assuming that continued eruption is a compensatory mechanism for facial growth, it seems reasonable to conclude that eruption follows the general pattern of craniofacial growth [1, 4].

The growth of the alveolar bone is less genetically determined compared to the rest of the skeleton and can be influenced by functional conditions and orthodontic treatment [7]. Accordingly, knowledge of the expected growth pattern of the alveolar bone is important in relation to orthodontic treatment of growing individuals. Furthermore, the morphology of the alveolar bone in three dimensions is important when it comes to the planning of orthodontic tooth movement as well as in connection with insertion of skeletal, temporary anchorage devises (TADs) and dental implants for restorative purpose [8–13].

There are, however, only limited reports where the craniofacial morphology has been used as a guideline for evaluation of height and width of the alveolar bone. A comprehensive thesis on the various facial anatomical components and the relationship between them was presented in 1966 by Solow [3]. In this thesis, a non-topographical positive correlation between height of the alveolar bone both in the maxilla and in the mandible, in relation to craniofacial height, was demonstrated in an adult sample. In more recent years, some studies have reported height and width of either maxillary or mandibular alveolar bone in relation to craniofacial height in dry skulls [14, 15] or patients [16–18].

Since its introduction in the late 1990s, cone beam computed tomography (CBCT) has become a common imaging modality that provides a three-dimensional data set of the facial skeleton. To our knowledge, only one study investigated the relationship between facial height and alveolar bone morphology in the incisor and molar regions of the maxilla and the mandible [19]. The aim of this study was therefore, based on CBCT images of subjects, to explore the association between the cross-sectional morphology of the maxillary and mandibular bodies at several sites of the tooth-bearing regions and craniofacial height.

## Methods

The study is conducted in accordance with the REporting of studies Conducted using Observational Routinely-collected health Data (RECORD) guidelines [20].

### Subjects

This study was conducted on pre-treatment CBCT scans collected from the archive at a private practice of orthodontics in Scandinavia. Images were obtained from patients, females over 15 years and males over 16 years, which were referred for specialist orthodontic and orthognathic treatment during 2008–2013.

CBCT scans from individuals with either missing permanent teeth other than third molars, periodontal disease visually detected on the radiographs, major asymmetries of the jaws or who had previously undergone orthodontic treatment were excluded.

### Radiography and categorisation of craniofacial height

Radiography was performed using an i-CAT CBCT (Imaging Sciences International, Hatfield, Pennsylvania, USA). The patients were seated in an upright position, with the head in natural head position. Field of view (FOV) was 16 × 13 cm, acquisition time was 8.9 s with a voxel size of 0.3 mm and exposure was set at 120 kVp and 18.54 mAs with a total radiation dose of 458.6 mGy cm^2^. Calibration of the i-CAT CBCT was performed according to the manufacturer’s requirements twice a year.

Lateral head images were generated from the CBCT scan using the i CAT software program. Cranio-facial height was determined cephalometrically using the Total Interactive Orthodontic Planning System (TIOPS) program [21].

**Fig. 1:**
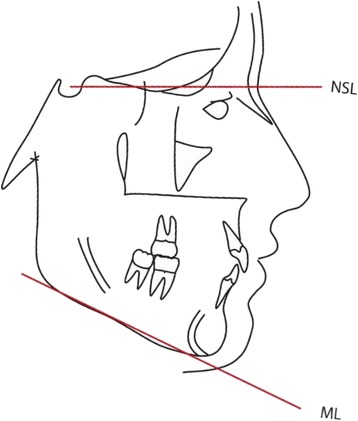
Cephalometric analysis. Landmarks and reference lines used for angular measurements in the lateral cephalograms, NSL nasion-sella line, ML mandibular line.

The measurement used was the inclination of the mandible in relation to the anterior cranial base. This is the angle formed between the nasion-sella line (NSL) and the mandibular line (ML), formed between menton and gonion (Fig. 1). The subjects were categorised in the three following groups: (i) a low angle (<27°), (ii) a normal angle (27–37°) and (iii) a high angle (>37°).

Scans from 450 patients were available from CBCT examinations performed during 2008–2013. After exclusion due to criteria previously stated, craniofacial height was cephalometrically determined for the rest of the scans. After identifying 60 scans from low-angle subjects, this number was set as the limit for the number of scans to be consecutively included in the normal- and high-angle groups for equal comparison. The number of individuals in each group was consequently set at 60 subjects.

By using iCATVision™ software [22], a fully reconstructed three-dimensional image with sagittal, coronal and axial slices was generated. When measuring in the maxilla, the nasion line (NL = a line from the anterior nasal spine continuing posteriorly through the hard palate/nasal floor to the posterior nasal spine) was used as the horizontal plane and upper limit for vertical measurements in the maxilla. When measuring in the mandible, the lower border of the mandible was repositioned horizontally prior to measurements. All measurements were performed by one of the authors who only had access to the decoded CBCT scans and blinded to all other patient information. Measurement values were simultaneously recorded in a statistical spread sheet by one of the other authors.

### Measurements of the cross-sectional maxillary and mandibular height and width

Three-dimensional images were reconstructed by the iCATVision™ software (version 1.7.0.7, Imaging Sciences International) and saved in digital imaging [22]. Generation of cross sections: A curved line was mapped to fit an axial CBCT section of the maxilla and mandible. Five cross sections (facial-lingual) were generated on each arch perpendicular to a tangent formed at these sites. Facial-lingual = *width* and vertical long axis = *height* **.**

**Fig. 2:**
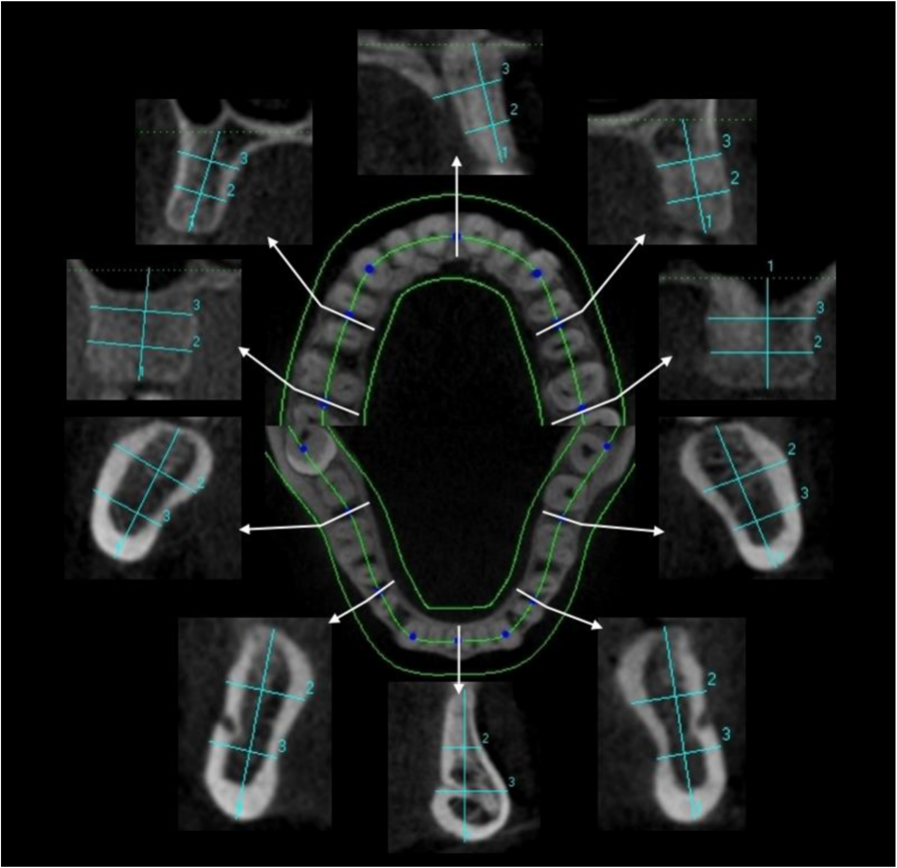
Cross-sectional bone measurements of the maxilla and mandible. One height and two width measurements at each site were measured. The sites were named according to location to the neighbouring teeth. I.e UP-MOL R upper molar right (side).

The height and width of the maxillary and mandibular cross sections where measured between the dentition at ten locations, five in the maxilla and five in the mandible. Each cross-sectional slide was measured at three sites, including one height and two width measurements (Fig. 2). The sites were named according to location in the following way: upper or lower jaw (UP or LO), molar (MOL), premolar (PRE) and midline (MID) and finally right (R) or left (L). Accordingly, UP-MOL-R stands for upper molar on the right side, height (H) (total length of the cross section), coronal width (W1) and apical width (W2) (Fig. 2).

The height was determined and measured by a line drawn through the long axis of the maxillary- or mandibular cross-sectional area. When measuring in the maxilla, the line was drawn perpendicular from the alveolar crest to the NL which was set as the upper limit. When measuring in the mandible, the line was drawn perpendicular from the alveolar crest to the ML. The height measurements then represented a total height, which included both alveolar bone and basal bone. The width measurements were recorded perpendicular to the long axis line at two locations. One line was drawn at a distance of one third and the other at two thirds of the alveolar length. Fifteen measurements were performed in the maxilla and mandible, respectively, giving a total of 30 measurements (Fig. 2).

Measurements were performed by one observer. To calculate intra-observer agreement, expressed as intra-class-correlation (ICC), the observer re-measured 20% of the sites in all the subjects after approximately 2 months. These sites were chosen by using random sampling.

### Statistical analysis

All statistical analysis was performed using IBM SPSS software (version 22.0; IBM Corp Armonk, NY, USA). For all variables, the three groups (low, normal, high) were compared using a one-way analysis of variance with a Tukey post hoc test. A significance level of 5% was used in all comparisons.

### Ethical approval

The study was conducted in accordance with the ethical principles of the World Medical Association Declaration of Helsinki (2008 version), approved by the Regional Ethical Review Board, Lund, Sweden (8 May 2014, Dnr 2014/288), the Danish Health and Medicines Authority, Denmark (20 July 2015, Sagsnr. 3-30-13-877/1/), and the Danish Data Protection Agency, Denmark (7 July 2015, J.nr. 2015-41-4117).

## Results

### Height measurements

**Fig. 3:**
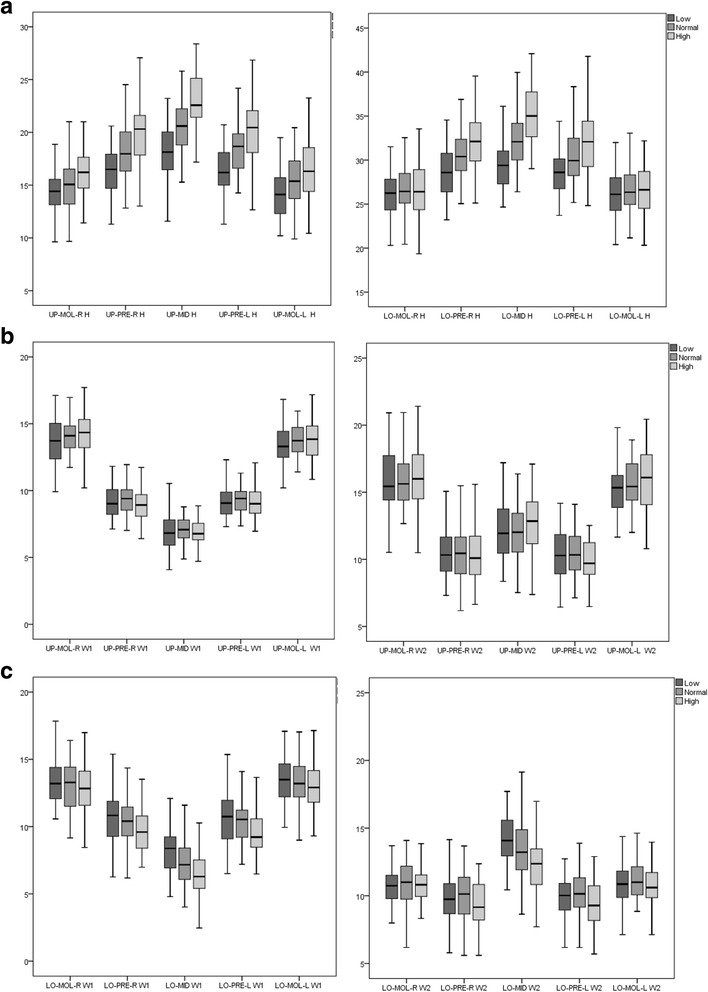
Cross-sectional height and width morphology measurements of the maxilla and mandible in the three groups (low-, normal- and high angle). *UP-MOL-R* (upper molar right), *UP-PRE-R* (upper premolar right), *UP-MID* (upper midline), *UP-PRE-L* (upper premolar left), *UP-MOL-L* (upper molar left), *LO-MOL-R* (lower molar right), *LO-PRE-R* (lower premolar right), *LO-MID* (lower midline), *LO-PRE-L* (lower premolar left), *LO-MOL-L* (lower molar left). *Y*-axis (mm). **a** Height (*H*) measurement at five cross-sectional sites in the maxilla and five in the mandible. **b** Width (*W*) measurement at five cross-sectional sites in the maxilla*. W1* demonstrates the coronal width measurements, and *W2* demonstrates the apical width measurements. **c** Width (*W*) measurement at five cross-sectional sites in the mandible*. W1* demonstrates the coronal width measurements, and *W2* demonstrates the apical width measurements. **a** Height in maxilla and mandible. **b** Coronal width. **c** Apical width

In both the *maxilla* and in the *mandible*, there was an increase in height from the molar region to the incisal region (midline) in all three groups. This increase was more pronounced in the high-angle group (displaying a steeper curve) and less pronounced in the low-angle group (a flatter curve) (Fig. 3a).

The midline area (UP-MID), the premolar area (UP-PRE) and the molar area (UP-MOL) of the *maxillary* cross section were significantly higher in the high-angle group compared to the normal- and the low-angle groups (Table 1).

**Table 1:** Bone height measurements of the maxillary and mandibular body in millimeters

	High-angle group			Normal-angle group			Low-angle group			Significance			
	$\overline{x}$	Range	SD	$\overline{x}$	Range	SD	$\overline{x}$	Range	SD	Overall	High-normal	Normal-low	High-low
Maxilla													
UP-MOL-R	16.2	9.6–23.6	2.5	15.1	9.7–21.0	2.4	14.3	9.6–21.0	2.2	0.000*	0.024*	0.169	0.000*
UP-PRE-R	20.0	13.0–27.1	2.7	18.2	12.8–24.5	2.4	16.3	11.3–20.6	2.1	0.000*	0.000*	0.000*	0.000*
UP-MID	22.9	15.3–28.4	2.7	20.6	15.3–25.8	2.3	18.3	11.6–26.1	2.7	0.000*	0.000*	0.000*	0.000*
UP-PRE-L	20.0	12.7–26.8	2.8	18.4	14.3–24.2	2.3	16.4	11.3–20.7	2.1	0.000*	0.002*	0.000*	0.000*
UP-MOL-L	16.3	10.4–23.3	2.6	15.4	9.9–20.4	2.5	14.1	10.2–19.5	2.2	0.000*	0.128	0.011*	0.000*
Mandible													
LO-MOL-R	26.7	19.4–36.6	2.6	26.8	20.43–33.5	2.6	26.2	20.3–31.5	2.6	0.455	0.994	0.492	0.558
LO-PRE-R	32.3	25.1–41.9	3.3	30.7	25.0–38.0	2.8	28.7	23.2–34.6	2.6	0.000*	0.011*	0.001*	0.000*
LO-MID	35.3	29.0–42.1	3.3	32.3	26.4–40.0	3.0	29.5	24.7–36.9	2.8	0.000*	0.000*	0.000*	0.000*
LO-PRE-L	32.1	24.8–41.8	3.6	30.7	25.2–38.3	3.0	28.7	23.7–34.4	2.6	0.000*	0.025*	0.001*	0.000*
LO-MOL-L	26.7	20.3–36.3	3.3	26.6	21.2–33.7	2.8	26.2	20.4–32.0	2.6	0.587	0.993	0.683	0.611

*UP-MOL-R* upper molar right, *UP-PRE-R* upper premolar right, *UP-MID* upper midline, *UP-PRE-L* upper premolar left, *UP-MOL-L* upper molar left, *LO-MOL-R* lower molar right, *LO-PRE-R* lower premolar right, *LO-MID* lower midline, *LO-PRE-L* lower premolar left, *LO-MOL-L* lower molar left, $\overline{x}$ mean value

*Statistically significant difference (*p* value < 0.05)

The midline area (LO-MID) and the premolar area (LO-PRE) of the *mandibular* cross section were significantly higher in the high-angle group compared to the normal- and the low-angle group. However, in the molar region, there were no significant differences between the groups (Table 1).

The range in height of the bone in the midline area was between 11.6 mm (low angle) and 28.4 mm (high angle) in the maxilla (Table 1).

The range in height of the bone in the midline area was between 24.7 mm (low angle) and 42.1 mm (high angle) in the mandible (Table 1).

### Width measurements

Concerning the cross-sectional coronal width measurements in the maxilla, Fig. 3b shows a V-shaped pattern, meaning that there was a decrease in width of the alveolar bone towards the midline area in all three groups. This decrease was more pronounced from the molar to premolar region for all three groups. Concerning the apical width measurement, the figure showed that the cross section was narrower in the premolar region compared to the molar and midline area (Fig. 3b).

Concerning the cross-sectional coronal width measurements in the mandible, Fig. 3c showed a V-shaped pattern, meaning that there was a decrease in width towards the midline area in all three groups. With reference to the apical width measurement, the figure showed that the cross section was significantly wider in the midline area compared to the premolar and molar area in all three groups (Fig. 3c).

There were no statistically significant differences in width measurements in the maxilla when comparing the three groups, neither in the coronal nor in the apical width measurements (Table 2).

**Table 2:** Bone width in millimeters of the maxillary and mandibular body

		High-angle group			Normal-angle group			Low-angle group			Significance			
		$\overline{x}$	Range	SD	$\overline{x}$	Range	SD	$\overline{x}$	Range	SD	Overall	Multiple comparisons		
												High-normal	Normal-low	High-low
Maxilla														
UP-MOL-R	W1	14.2	10.2–17.7	1.6	14.1	11.7–17.6	1.3	13.7	9.9–17.1	1.5	0.263	0.953	0.425	0.272
	W2	16.1	10.5–21.4	2.4	15.9	12.7–21.9	1.9	15.9	10.5–21.0	2.1	0.899	0.913	1.000	0.919
UP-PRE-R	W1	9.0	5.5–11.7	1.3	9.3	7.0–11.9	1.1	9.2	7.13–11.8	1.2	0.239	0.208	0.655	0.690
	W2	10.4	6.6–16.4	2.2	10.4	6.2–15.5	1.8	10.6	7.3–15.9	2.0	0.815	0.976	0.908	0.804
UP-MID	W1	6.9	4.8–10.5	1.4	7.1	4.9–8.8	1.0	6.9	4.1–10.5	1.4	0.631	0.652	0.721	0.993
	W2	12.6	7.4–17.1	2.1	12.0	7.5–16.4	2.5	12.3	8.4–19.3	2.5	0.262	0.231	0.724	0.657
UP-PRE-L	W1	9.0	5.7–12.1	1.2	9.3	6.2–12.3	1.1	9.1	7.3–12.4	1.2	0.539	0.508	0.873	0.813
	W2	10.0	6.5–16.3	1.9	10.4	7.1–14.1	1.7	10.5	6.4–17.1	2.0	0.345	0.458	0.990	0.381
UP-MOL-L	W1	13.8	10.8–18.1	1.7	13.8	11.4–16.0	1.2	13.4	10.2–16.8	1.3	0.175	0.994	0.261	0.218
	W2	15.9	10.2–20.4	2.1	15.9	12.0–18.9	1.8	15.3	10.2–19.8	1.9	0.292	0.728	0.694	0.259
Mandible														
LO-MOL-R	W1	12.8	7.5–17.0	1.9	13.0	9.2–16.4	1.9	13.2	6.8–17.8	2.0	0.460	0.730	0.879	0.432
	W2	10.8	6.3–14.3	1.6	11.0	6.2–14.1	1.6	10.7	6.2–14.6	1.6	0.661	0.817	0.646	0.956
LO-PRE-R	W1	9.7	7.0–13.5	1.7	10.4	5.1–15.8	2.1	10.6	6.3–15.4	1.9	0.035*	0.138	0.824	0.035*
	W2	9.4	5.6–12.4	1.7	10.0	5.6–13.7	1.8	9.8	5.8–14.2	1.7	0.122	0.111	0.801	0.352
LO-MID	W1	6.4	2.5–10.3	1.5	7.3	4.0–11.6	1.6	8.2	4.8–12.1	1.6	0.000*	0.004*	0.006*	0.000*
	W2	12.3	7.7–17.0	2.1	13.5	8.6–22.8	2.4	14.1	7.3–17.7	1.9	0.000*	0.005*	0.282	0.000*
LO-PRE-L	W1	9.5	6.5–13.7	1.6	10.4	5.8–15.5	2.0	10.5	6.5–15.4	2.0	0.005*	0.025*	0.914	0.008*
	W2	9.4	5.7–12.9	1.8	10.2	6.2–13.9	1.7	10.0	6.2–14.6	1.7	0.045*	0.045*	0.833	0.164
LO-MOL-L	W1	13.0	7.0–17.1	1.9	13.3	9.0–17.0	1.9	13.3	6.2–17.1	2.0	0.528	0.677	0.970	0.529
	W2	10.6	7.0–14.0	1.4	11.0	5.4–14.6	1.7	10.9	7.1–14.9	1.6	0.812	0.420	0.888	0.706

W1 demonstrates the coronal width measurements, and W2 demonstrates the apical width measurements. *Y*-axis (mm)

*UP-MOL-R* upper molar right, *UP-PRE-R* upper premolar right, *UP-MID* upper midline, *UP-PRE-L* upper premolar left, *UP-MOL-L* upper molar left, *LO-MOL-R* lower molar right, *LO-PRE-R* lower premolar right, *LO-MID* lower midline, *LO-PRE-L* lower premolar left, *LO-MOL-L* lower molar left, $\overline{x}$ mean value

*Statistically significant difference (*p* value < 0.05)

In the coronal and apical midline area in the mandible (LO-MID), the bone was significantly narrower in the high-angle group compared to the normal- and the low-angle groups. In the molar area, there were no statistically significant differences in the coronal or in the apical width measurements between the three groups (Table 2).

The range in width, of the cross sections in the coronal midline area, was between 4.1 mm (low angle) and 10.5 mm (high angle/low angle) in the maxilla (Table 2).

The range in width of the coronal part of the cross section in the midline area was between 2.5 mm (high angle) and 12.1 mm (low angle) in the mandible (Table 2).

### Gender differences

In a majority of the cross-sectional measurements (73%), significant differences were evident; male subjects tended to have wider and higher cross sections compared to female subjects. Males displayed approximately 1 mm larger dimension of the maxillary and mandibular bone both in vertical and horizontal measurements (Table 3).

**Table 3:** Difference between male and female when comparing measurements of jaw bone height and width in millimeters

		Male		Female	
Maxilla					
UP-MID	Height	21.0		20.4	
	Width I	7.5*		6.7*	
	Width II	13.4*		11.7*	
		Right	Left	Right	Left
UP-PRE	Height	19.0*	19.1*	17.7*	17.8*
	Width I	9.7*	9.7*	8.9*	8.8*
	Width II	11.0*	10.9*	10.2*	9.9*
UP-MOL	Height	16.4*	16.5*	14.6*	14.6*
	Width I	14.7*	14.3*	13.6*	13.3*
	Width II	17.0*	16.4*	15.4*	15.1*
Mandible					
LO-MID	Height	34.6*		31.2*	
	Width I	7.4		7.2	
	Width II	14.0*		12.9*	
		Right	Left	Right	Left
LO-PRE	Height	32.2*	32.4*	29.7*	29.5*
	Width I	10.6*	10.7*	10.0*	9.9*
	Width II	10.0	10.1	9.6	9.7
LO-MOL	Height	28.3*	28.3*	25.6*	25.6*
	Width I	13.3	13.4	12.9	13.0
	Width II	10.8	10.7	10.9	10.9

Right side, left side, midline (only one/jaw). Width I: The coronal width measurements. Width II: The apical width measurements

*UP-MID* upper midline, *UP-PRE* upper premolar, *UP-MOL* upper molar, *LO-MID* lower midline, *LO-PRE* lower premolar, *LO-MOL* lower molar

*Statistically significant difference (*p* < 0.05)

### Intra-observer agreement

One thousand eighty (20%) out of 5400 measurements were randomly selected for re-measurement, and intra-class correlation (ICC) was calculated for intra-observer agreement. The correlation in height measures were between 0.858 and 0.989. The correlation in width I measures were between 0.762 and 0.944, and the correlation in width II measures were between 0.696 and 0.923.

## Discussion

As CBCT is a rather new imaging modality, introduced in the late 1990s, there were no European guidelines on the use of CBCT for different dental- and maxillofacial conditions at the time when the majority of images used in this study were obtained. In 2012, European guidelines for the use of CBCT were published [23] and there is a continuous interest in stating precise selection criteria following the principle of As Low As Reasonable Achievable (ALARA) for the use of X-rays [24]. The selection criteria for performing CBCT were craniofacial- and skeletal deviations, impacted teeth, patients planned for orthognathic surgery and trauma patients, none of which can be specifically disputed. It might be, however, if a study like this should be performed prospectively that selection criteria might be adjusted. Having access to these retrospectively collected images, from which a selection was used for this study, we considered it unethical not to obtain clinically valuable data. Ethical approval was obtained for the use of these available CBCT images for research purpose.

Craniofacial height was determined cephalometrically using the Total Interactive Orthodontic Planning System (TIOPS) program [21] by one of the authors, a specialist in orthodontics. This analysis is a standard tool in orthodontic assessment and treatment planning. Observer reliability of this technique has shown to be high, both in conventional lateral radiographs and in CBCT scull images [25, 26]. Height and width measurements of the maxillary and mandibular bodies was repeated at 20% of the sites showing a moderate to almost perfect agreement interpreted according to Landis and Koch [27].

Craniofacial morphogenesis evolves through an interaction between the development of the facial tissue and that of the supporting skeletal framework. In the present study, focus has been on an aspect of craniofacial morphogenesis, which is known as the dentoalveolar compensatory mechanism. The present study indicates that in a high-angle craniofacial pattern, the bone in the mandibular midline is high and narrow, whereas in the low-angle morphology, the bone is shorter and wider.

### Maxillary height

Gracco et al. showed no statistical difference in the midline area of the maxilla between high-, normal- and low-angle subjects on CBCT images. However, they investigated only the anterior region of the maxilla [16]. These results are not in agreement with our findings, which could be due to that growing individuals (12–40 years) were part of the study and some changes of the morphology of the maxillary bone must be anticipated to occur as the individuals grow [16]. The differences could also be due to a slightly different measuring technique. In a recent investigation by Sadek et al. [19], a significant difference in height of the maxillary cross-sectional sites in the midline area was also reported. However, no significant differences were found in the molar region.

### Mandibular height

The subjects with the high-angle morphology revealed a significant higher mandibular cross section in the midline and premolar region when compared to the other groups. These results are in accordance with Sadek et al. [19] and almost in accordance with the findings by Swasty et al. [18]. The only difference was that Swasty et al. demonstrated a negative correlation of the mandibular body in the molar region, where the high-angle group had a shorter body in the molar region compared to the low-angle group [18]. The same feature was also described by Kohakura et al. [28]. However, in the present investigation, we could not demonstrate a statistical difference in height in the molar region when comparing the groups. The difference between the findings can be explained by the fact that individuals who were still growing were included in the Swasty study [18].

In our study as well as in Swasty et al. and Sadek et al. [18, 19], a pronounced difference in height from the molar to the midline region was described in the high-angle group and the least change in height proceeding from the molars to the midline was displayed in the low-angle group. Considering the growth pattern of the mandible, these observations reflect the rotation of the mandible, where the incisors have to erupt significantly in order to compensate for the posterior rotations and the molars have to erupt less, whereas the incisors have to erupt less in subjects with an anterior rotating mandible [5].

### Maxillary width

No significant difference was found in width measurements in the maxilla when comparing the three groups, neither in the coronal nor in the apical width measurements in the present study. This is not in accordance with the results reported by Gracco et al. [16] who described a negative correlation in width measurements in the midline region between subjects with different vertical craniofacial dimensions. In the study by Sadek et al. [19], significant difference between high- and low-angle individuals in width was reported in the midline area both in the coronal and apical region. The reasons for this difference could be the following: (1) they did not measure between the teeth, but across the incisors, and (2) the measurements were obtained at the mid-root level and at the apex making the measurements dependent more on the lengths of the root than on the length of the alveolar bone. Since the length of the bone seems to vary more than the length of the root, which is a more stable reference point, the cross-sectional measurements will not be equal.

### Mandibular width

Figure 3c demonstrated a V-shaped pattern when presenting the measurements of the coronal region, as the width decreased along the dental arch towards the midline. The same feature was described by Swasty et al. [18]. In the alveolar coronal region, the mandible was significantly thinner in the midline and premolar region in all three groups. This is in accordance with the results of both Swasty et al. and Sadek et al. [18–19]. Concerning the apical measurement, the cross section was significantly wider in the midline area compared to the premolar and molar area in all three groups (Fig. 3c). This could be due to the anatomy of the mental spine and the base of the mandible in the front region.

### Gender differences

A general pattern was displayed, where the males presented a greater height and width compared to the females. Swasty et al. [18] described that there was no statistical differences in cortical bone thickness between the genders. However, they found a statistical difference in height of the cross sections between males and females. Accordingly, it seems reasonable to conclude that at least in height of the bone, there seems to be a gender-correlated difference.

### Clinical implications

The results of this investigation may support the fact that the dentoalveolar compensatory mechanism aims to maintain a functional occlusion in connection with craniofacial growth. In the high-angle group, the alveolar bone had to grow more in order to compensate or partly compensate for the vertical craniofacial growth, and therefore, the bodies of the maxilla and mandible became higher. The fact that the arches became narrower in the midline of the mandible might be due to the difference in loading. It can be hypothesised that the alveolar bone in the high-angle subjects are less loaded than the bone in the low-angle group. In the low-angle group, the root resides in most of the arch, whereas in the high angle group, the root resides only in part of the body, which according to Wolff’s law will result in a less developed bone [29].

In orthodontic treatment, it is very important to understand how the development of the lower third of the face is closely linked to the dentoalveolar compensatory mechanism, because the dentoalveolar compensatory mechanism can be influenced in connection with treatment.

Also during active orthodontic treatment, knowledge of the morphology of the alveolar bone is important. In thin ridges, the buccal and lingual cortices can contact or be in close approximation with very little cancellous bone. The risk for root resorption increases if the roots are torqued into the cortex. In addition, there is a risk for moving teeth out of alveolar bone that has a narrow width [30].

In connection with insertions of skeletal TADs, the difference in the morphology of the cross section of the maxillary and mandibular bodies in different growth patterns is valuable information for therapy planning and adequate therapy. It has been proven that in many situations, the palate is an ideal area for screw insertion where the screw is inserted at the third rugae with the tip in an anterior direction [9–11]. However, knowledge of the anatomy at the insertion site is important, and the present study has demonstrated that the alveolar bone is short in the mid-palatal area in the low-angle face compared to the normal- and high-angle cases, with less space superior to the apex of the incisors. Accordingly, an increased risk of root damage and insertion of the screw into the nasal cavity can be expected in low-angle patients.

Likewise, during treatment planning for growing patients with congenital missing teeth, the decision to replace missing teeth with implants or by orthodontic closure could be influenced by the expected development of the dentoalveolar compensatory mechanism. The risk of infra-occlusion of the implant supported crowns might be higher in the high-angle group, where the incisors seem to erupt more.

Vertical control is a common treatment approach in high-angle cases, and appliances such as skeletal anchorage devices are described as very useful [31]. However, considering the A-shaped morphology (Fig. 3a) of the maxillary body in the vertical dimension, this treatment approach will actually accentuate the difference in height between the molar and incisor region. In orthognathic surgery, the aim is to increase the vertical dimension in the posterior region, which will flatten the same curve. How this difference in treatment strategies, aiming to solve the same problem, will influence final stability, airways and function should be investigated further.

In treatment with dental implants, a careful preoperative evaluation is important in order to achieve a predictable, successful aesthetic outcome. The buccal bone wall thickness is of crucial importance when it comes to selection of appropriate treatment approach [32]. The overall width of the mandibular body in a high-angle subject tend to be thinner, which probably gives a thinner alveolar bone. In the present study, the range in width of the coronal part of the cross section in the midline area was between 2.5 mm (high angle) and 12.1 mm (low angle) in the mandible (Table 2). As suggested by Buser et al., to achieve an aesthetic and functional success in dental implant treatment, it is important to take into account the three-dimensional aspect of the alveolar bone [13]. It is recommended that the dental implant should be placed inside the biological envelope of the alveolar bone in order to obtain the best conditions for success [8].

## Conclusions

The main findings of the present study were:

(1) The high-angle group had a significantly longer cross-sectional area of the maxillary and mandibular body compared to the other two groups.

(2) The high-angle group also had significantly narrower mandibular cross-sectional body compared to the other two groups, especially in the anterior region.

In this retrospective study, it has been demonstrated that there is a close link between vertical craniofacial development and the morphology of cross-sectional sites of the maxillary and mandibular bodies for patients referred for orthodontic and orthognathic treatment. Understanding this biological link is important in connection with many comprehensive dental- and orthodontic treatments.
